# Composite Fibers from Recycled Plastics Using Melt Centrifugal Spinning

**DOI:** 10.3390/ma10091044

**Published:** 2017-09-06

**Authors:** Nicole E. Zander, Margaret Gillan, Daniel Sweetser

**Affiliations:** United States Army Research Laboratory, Weapons and Materials Research Directorate, Aberdeen Proving Ground, Aberdeen, MD 21005, USA; margaret.gillan2.ctr@mail.mil (M.G.); sweetser@udel.edu (D.S.)

**Keywords:** melt spinning, centrifugal spinning, recycled polymers, polyethylene terephthalate, polystyrene, polypropylene, DSC, FTIR, tensile strength

## Abstract

New methods are being developed to enable the production of value-added materials from high-volume, low-cost feedstocks arising from domestic recycling streams. In this work, recycled bottle-grade polyethylene terephthalate, polystyrene, and polypropylene were spun into fibers from the melt using a centrifugal spinning technique. Mono-component fibers and 50/50 blends of each polymer and a 33/33/33 blend of all three polymers were evaluated. Fiber morphology, chemistry, thermal, and mechanical properties were probed. Fiber diameters ranged from ca. 1 to over 12 µm, with polypropylene fibers having the smallest fiber diameters. Mono-component fibers were generally defect-free, while composite fibers containing polypropylene were beady. Fibers made from polyethylene terephthalate had the highest tensile strength, and the addition of polyethylene terephthalate to the other polymers improved the mechanical properties of the blends. Nano- and micro-fibers from both pure and mixed waste streams are expected to have applications in myriad areas such as ultra/micro-filtration, composites, and insulation.

## 1. Introduction

Of the nearly 32 million tons of plastics generated in the U.S. each year, only about 5% are recycled [1,2]. Currently, the value of recycled plastics is low and the cost for recycling is not always economically viable, although the environmental benefits are high. Until alternate reprocessing methods and higher-value uses are developed, the recycling trends are not expected to change. New low-cost reprocessing methods are particularly needed in places such as many Forward Operating Bases (FOBs) that do not have the required infrastructure for recycling.

Recent reports have shown the feasibility of turning recycled plastics into nanofibers [3,4,5]. Nanofibers, known for their high surface area to volume ratios and potentially superior mechanical performance over macro-scale fibers, represent a potential value added application for waste plastics. Nanofibers are used in a wide variety of research and commercial areas including filtration, composites, tissue culture, and medical materials. Nanofibers are commonly prepared using electrospinning, a process that uses high voltage and generally requires organic solvents. The fibers are formed as the polymer solution emerges from a charged needle, where coulombic repulsion causes the spin solution to form a spray of jets that simultaneously dry and draw the polymer nanofiber. Polystyrene nanofibers from styrofoam and a non-toxic solvent d-limonene were fabricated using electrospinning by Shin and Chase et al., while Shin et al. used common organic solvents [4,5]. Bottle-grade PET (polyethylene terephthalate) nanofibers from post-consumer bottles were generated by Rajabinejad et al. using melt-electrospinning [6]. 

While electrospinning is extremely versatile and requires minimal equipment (low cost), the electrospinning process suffers from low production rates (typically 0.2 g h^−1^ for lab scale), has a minimal solvent-less melt-spinning capability, and is limited to soluble polymers [7]. Centrifugal spinning is a relatively new technique that overcomes many of the drawbacks of electrospinning [8,9,10,11]. Centrifugal spinning uses a heated spinneret that melts and elongates the polymer jet using centrifugal force rather than high voltage, enabling nano- to micro-fiber formation with non-conductive and insoluble polymers via this process [8]. Production rates are significantly higher, with values of up to 300 g h^−1^ for a Forcespinning™ benchtop unit (Fiberio, McAllen, TX, USA). The range of polymers that can be spun using this process is just beginning to be explored. Badrossamay et al. spun fibers from gelatin, polyethylene oxide, and polylactic acid solutions [10]. Senthilram and McEachin spun polycaprolactone from solution [7,12]. Fang et al. generated fibers from monomer solutions using thiol-ene photopolymerization coupled with centrifugal spinning [13]. Fibers have also been prepared from the melt using centrifugal spinning. In our previous work, polycaprolactone fibers were prepared at varied rotation speeds and melt temperatures to understand the effect on fiber morphology and crystallinity [11]. Ragahavan et al. fabricated melt spun polypropylene fibers, while Sarkar et al. generated melt spun polystyrene fibers [9,14].

With the increased use of plastics in consumer products and packaging, the need for re-use options is ever growing. Mechanical recycling, or reprocessing plastic waste into secondary products by physical means, generally results in products with mediocre mechanical properties. There are two main factors that lead to the reduced performance of recycled plastic, including degradation of the plastic due to high temperature processing and shearing, and ageing and heterogeneity of the plastic waste. The waste is a mixture of various grades and types of polymers, most of which are often incompatible, and may contain contaminants such as paper, adhesives, and other additives which reduce the performance of the recycled products. The complete separation of components, particularly different molecular weights of the same polymer, and different chemical compositions, is rarely implemented [15]. Reprocessing of polymers can lead to viscosity changes from degradation processes such as chain scission, branching, or crosslinking, and changes in crystallization behavior [16].

Polyethylene terephthalate (PET) waste is an abundant resource due to the popularity of disposable food and drink packaging. In the United States alone, about 2 million tons per year are wasted (not recycled). Post-consumer bottles are regarded as one of the most important waste materials due to their tensile and impact strengths, chemical resistance, processability, and thermal stability. The bottle caps and labels are typically made from polypropylene (PP) and account for 5–10% of the total bottle weight. The aforementioned polymers are easily melted and processed into micro-fibers with a commercial Forcespinning™ benchtop system. In this work, in addition to processing PET, polypropylene (PP), and polystyrene (PS) into fibers, equal part blends of each combination of polymers (50/50 wt % of PS, PET, PP), as well as a tri-component blend of PS, PET, and PP (33/33/33 wt %) were prepared. The effect of rotation speed and blend composition were evaluated to understand which compositions yield fibers with the highest tensile strength and elastic modulus. These properties are important for two common applications of fibrous materials: filtration and composite reinforcement.

## 2. Results

### 2.1. Fiber Morphology

**Figure 1:**
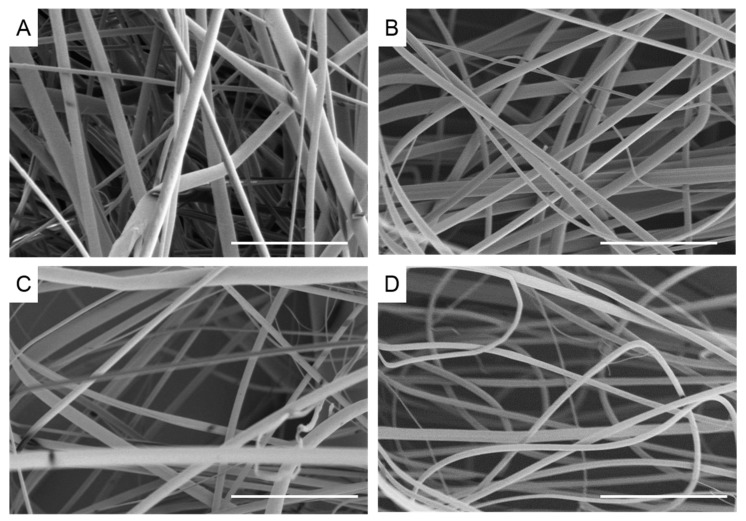
Scanning electron micrographs of recycled polyethylene terephthalate fibers melted at 300 °C at different rotation speeds (**A**) 6000 rpm; (**B**) 8000 rpm; (**C**) 10,000 rpm; (**D**) 12,000 rpm. Scale bar denotes 100 µm.

**Figure 2:**
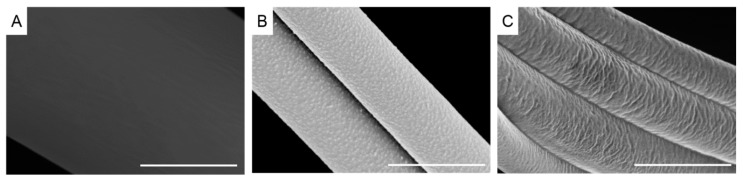
Scanning electron micrographs of fiber surfaces (**A**) recycled polyethylene terephthalate; (**B**) recycled polystyrene; (**C**) recycled polypropylene. Scale bar denotes 4 µm.

Micrometer sized fibers were formed using the centrifugal spinning process from the melt. Rotation speed was varied between 6000 and 12,000 rpm in order to examine the effect on fiber diameter and morphology. Figure 1 displays SEM images of recycled PET (rPET) fibers. The fibers had a smooth surface, as can be seen in the high resolution image in Figure 2A. The fibers are nearly defect and bead free at all rotation speeds, although there is a fairly wide distribution in fiber diameters. The fiber diameter was larger for the fibers formed at the slowest rotation speed (9.41 ± 4.2 µm, *n* = 252) and was significantly reduced to 5.84 ± 3.1 µm at 8000 rpm (*n* = 353) (Table 1). The fiber diameter was further reduced at 10,000 rpm, but was not significantly different than the fibers spun at 12,000 rpm (4.68 ± 4.0 µm (*n* = 313) and 4.56 ± 3.0 µm (*n* = 377), respectively).

**Figure 3:**
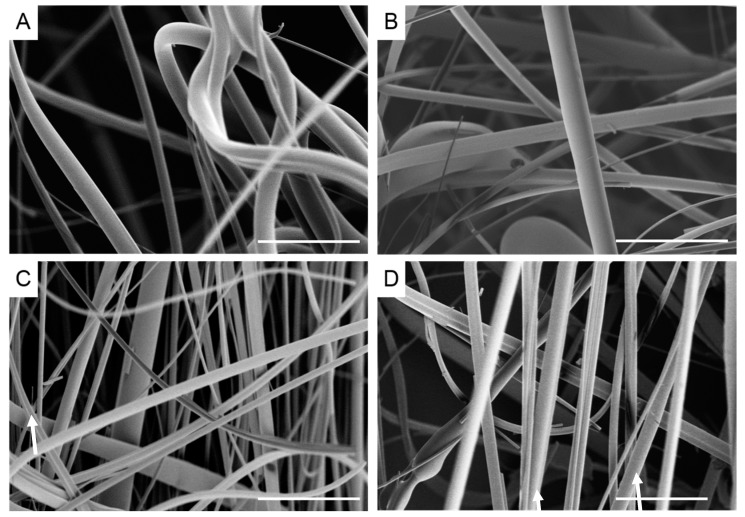
Scanning electron micrographs of recycled polystyrene fibers melted at 260 °C at different rotation speeds (**A**) 6000 rpm; (**B**) 8000 rpm; (**C**) 10,000 rpm; (**D**) 12,000 rpm. Scale bar denotes 100 µm. Arrows denote broken fibers.

Images of recycled polystyrene (rPS) fibers are displayed in Figure 3. The fibers had a rough surface, possibly due to partial degradation of the polymer (Figure 2B). Fibers exhibited some beads and there was evidence of fiber breakage, likely occurring during fiber spinning. Beading did not appear to be correlated with rotation speed or temperature, while increased fiber breakage was observed at higher rotation speeds. Fiber diameters were significantly reduced as the rotation speed was increased (8.10 ± 5.3 µm (*n* = 178, 6000 rpm), 6.13 ± 5.2 µm (*n* = 330, 8000 rpm), 4.74 ± 3.1 µm (*n* = 329, 10,000 rpm), 3.00 ± 3.0 µm (*n* = 151, 12,000 rpm)) (Table 1).

**Figure 4:**
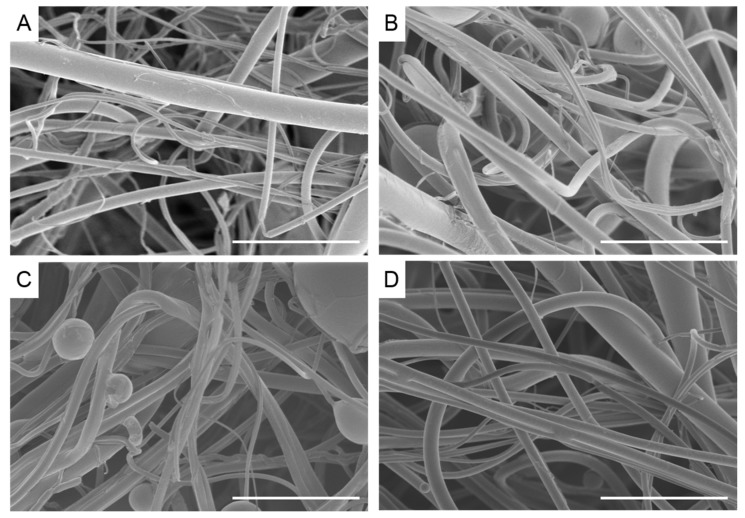
Scanning electron micrographs of recycled polypropylene fibers melted at 230 °C at different rotation speeds (**A**) 6000 rpm; (**B**) 8000 rpm; (**C**) 10,000 rpm; (**D**) 12,000 rpm. Scale bar denotes 50 µm.

Recycled polypropylene fibers (rPP) (Figure 4) exhibited a fair amount of beading, particularly at the higher rotation speeds due to the relatively low viscosity compared to rPS and rPET (discussed in a subsequent section). Fiber diameters were also smaller than rPS and rPET fibers for this reason (Table 1). The surface of the fibers exhibited a shark skin morphology, formed from melt flow instability, possibly due to slippage at the orifice wall (Figure 2C) [17]. Polypropylene was the only polymer with a low enough melt viscosity to form fibers using the smaller orifice spinneret (30 gauge). Fiber diameters were significantly reduced with an increased rotation speed (5.98 ± 2.9 µm (*n* = 187, 6000 rpm), 3.93 ± 2.4 µm (*n* = 185, 8000 rpm), 2.65 ± 1.6 µm (*n* = 105, 10,000 rpm), 1.92 ± 0.9 µm (*n* = 96, 12,000 rpm)). Fiber diameters with the 30 gauge spinneret were further reduced, approaching nanometer dimensions (2.09 ± 1.3 µm to 1.07 ± 0.7 µm).

### 2.2. Composite Fiber Morphology

**Figure 5:**
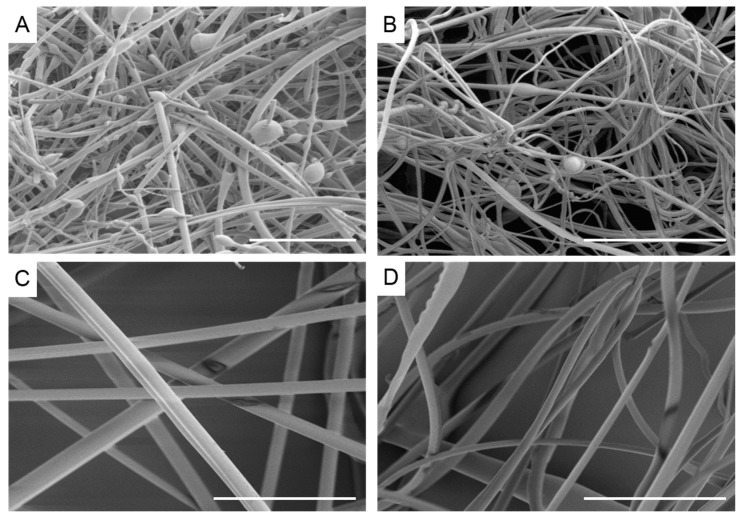
Scanning electron micrographs of recycled fibers melted at 270 °C 10,000 rpm (**A**) 50/50 wt % polypropylene/polystyrene; (**B**) 50/50 wt % polypropylene/polyethylene terephthalate; (**C**) 50/50 wt % polystyrene/polyethylene terephthalate; (**D**) 33/33/33 wt % polystyrene/polypropylene/polyethylene terephthalate. Scale bar denotes 100 µm.

**Figure 6:**
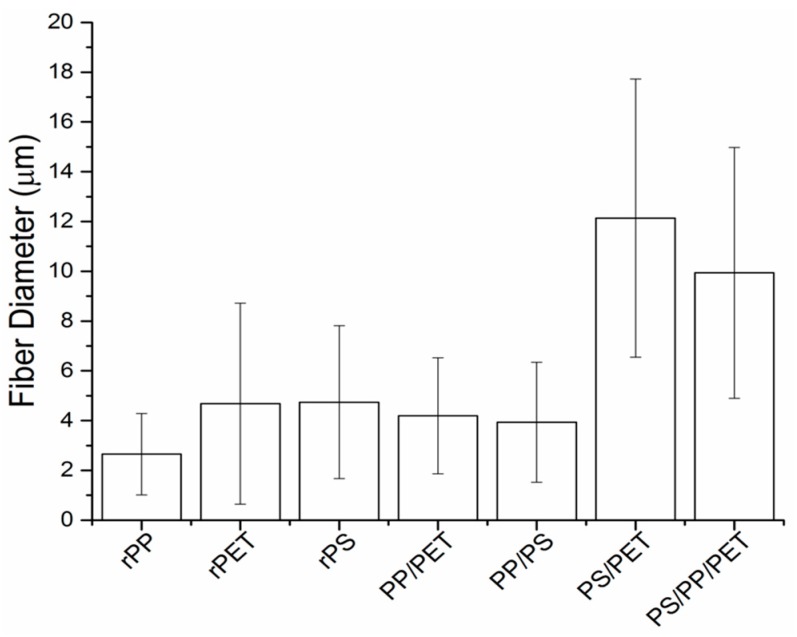
Fiber diameter of recycled polymers and blends (50/50 and 33/33/33 wt %) at 10,000 rpm rotation speed. Polymers were melt-spun at the following temperatures: polypropylene (PP) = 230 °C, polystyrene (PS) = 260 °C, polyethylene terephthalate (PET) = 300 °C, blends = 270 °C. Error bars correspond to the standard deviation calculated from three to five replicates of each polymer.

Blends of rPET, rPS, and rPP (denoted PP/PS, PP/PET, PS/PET, and PS/PP/PET) were prepared to test the feasibility of generating fibers from mixed waste streams, and thus minimize plastic sorting requirements. Images of the binary (50/50 wt %) and ternary (33/33/33 wt %) blends are displayed in Figure 5. The PP/PS blend resulted in beady fibers, as well as a number of broken fibers (Figure 5A). The PP/PET fibers had less beads but were still not defect free (Figure 5B). The beads in the composite fiber mats containing PP were likely due to the low viscosity of PP compared to PS and PET. Although the overall viscosity of the melt increased with the addition of PS or PET (discussed in a subsequent section), the viscosity of PP may have remained mostly unchanged due to its immiscibility with PS and PET. The PS/PET and PS/PP/PET fibers were much larger than the other binary blend fibers but were defect free (Figure 5C,D). The fiber diameters for the PP/PET and PP/PS blends were similar to the PS and PET fibers (Figure 6). The diameters of the PS/PET and PS/PP/PET fibers were two to three times larger than the other fibers. 

### 2.3. Viscosity

**Figure 7:**
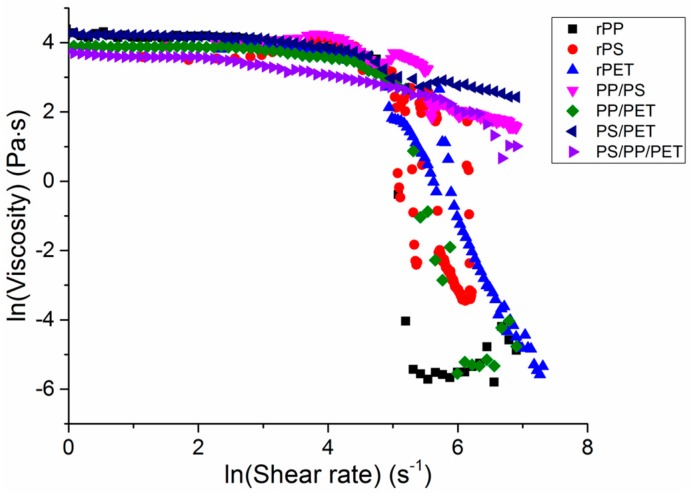
Rheological behavior of recycled polymers and blends (50/50 wt % and 33/33/33 wt %). Experiments were conducted at the melt-spinning temperatures for each polymer (polypropylene (PP) = 230 °C, polystyrene (PS) = 260 °C, polyethylene terephthalate (PET) = 300 °C, blends = 270 °C).

The viscosity of the single polymer melts and blends was probed to understand the effect of the melt composition on viscosity (Figure 7). At low shear rates, the polymer melts had similar viscosities and behaved as Newtonian fluids. At shear rates above 8 s^−1^, a power-law behavior, in which shear thinning occurs, was observed. The viscosity of all polymers decreased quickly at shear rates of ca. 148 s^−1^. In general, the viscosity correlated well with observed fiber diameters, which has been well documented, particularly in the electrospinning literature [18]. All of the blends with the exception of PP/PET had the highest viscosities and corresponding fiber diameters, followed by PET and then PS, PP/PET, and PP.

Shear rates during the melt spinning process ranged from 100 s^−1^ to 200 s^−1^, or generally in the shear-thinning range. Elamri et al., Kale and Bopardikar, and Nichetti and Manas-Zloczower found similar reductions in viscosity at these shear rates for bottle-grade PET, PP, and PS, respectively [19,20,21]. Higher rotation speeds led to increased fiber drawing and a resulting decrease in fiber diameter, as seen in Table 1.

### 2.4. Chemical Analysis

**Figure 8:**
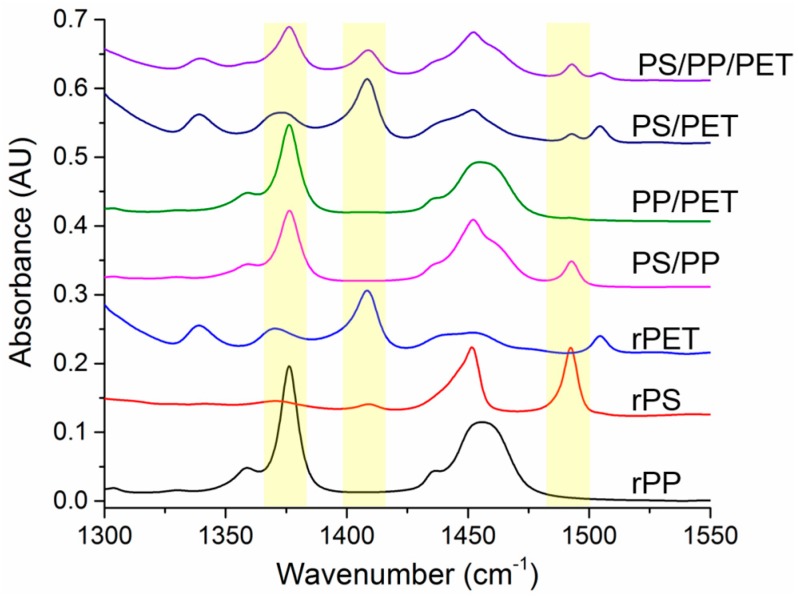
FTIR spectra of recycled polymers and blends (50/50 wt % and 33/33/33 wt %). Characteristic regions of each polymer are highlighted: polypropylene (PP), polystyrene (PS), polyethylene terephthalate (PET).

The spun fibers were examined using FTIR to verify the fiber composition. Non-overlapping peaks for each polymer were identified and are highlighted in Figure 8. Full range spectra have been added to the Supplementary Materials section (Figure S1). The CH_3_ band at 1376 cm^−1^ was utilized for PP, while the aromatic C=C stretching bands were used to identify PS (1490 cm^−1^) and PET (1409 cm^−1^). The PP identifier peak is only present in the pure fibers and the PS/PP, PP/PET, and ternary blend fibers. Likewise, the PS and PET identifier peaks are only present in the expected fibers. The presence of all expected polymers were verified, indicating that the different polymer types were all incorporated into the spun fibers. 

The relative composition of the fiber mats was determined by integrating the identifier peaks for each polymer and normalizing them to a peak (1720 cm^−1^) present in PP, PS, and PET. The fiber mats containing PP had relatively higher amounts of this polymer than was expected. The PP/PS blend fibers had 84% PP, the PP/PET blend fibers had 40% PP, and the ternary blend fibers had 53% PP (20% PS, 27% PET). This is likely due to the lower viscosity of the PP compared to the other polymers. PET appeared to extrude from the spinneret better than the PS, although PET was more viscous. The PS/PET blend fibers were composed of 93% PET. The PS (and PP) were able to be ground into a fine powder, whereas the PET consisted of fluffy shreds. Since the materials were only hand mixed (right before charging the spinneret), it is possible that more PET could have been loaded into the spinneret because the PS or PP powder may have settled into the bottom of the jar. 

### 2.5. Mechanical Testing

**Figure 9:**
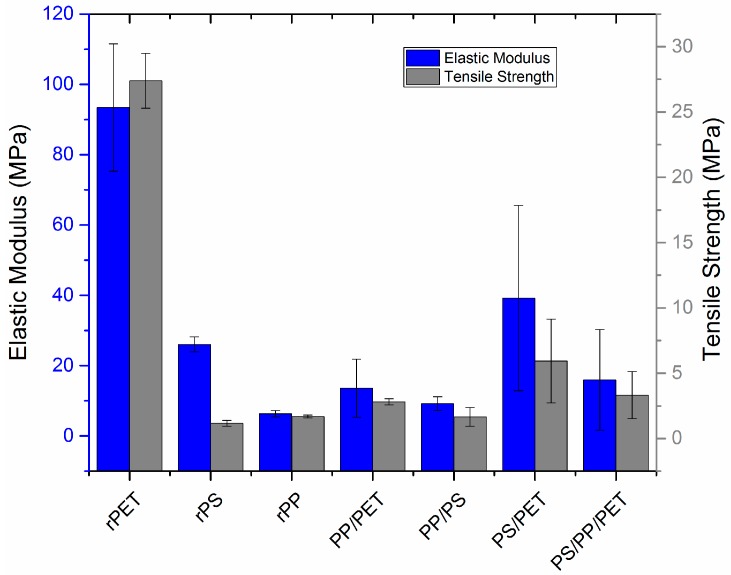
Elastic modulus and tensile strength of recycled fibers as determined by uniaxial tensile testing. PP = polypropylene, PS = polystyrene, PET = polyethylene terephthalate. Blends consisted of either 50/50 wt % or 33/33/33 wt % of each polymer as noted. Error bars correspond to the standard deviation calculated from three to five replicates of each polymer.

Uniaxial mechanical characterization was carried out using a microtensile stage (Figure 9). Representative load-displacement curves are displayed in the Supplementary Materials section (Figure S2). PET fibers had the highest elastic modulus and tensile strength, followed by blends with PET with the exception of the PP/PET blend. PET and PP fibers stretched to 70–80% elongation before failing, whereas the PS fibers were quite brittle and failed at less than 10% elongation. The addition of PS to PP did not impact elongation to failure; PP/PS fibers stretched to ca. 70–80% before failing. However, the addition of either PP or PS to PET reduced elongation to 30% and 40%, respectively. The ternary blend fibers were quite brittle and failed at 20% elongation. Note that fiber diameter was not uniform for all the samples tested, but varied according to Figure 6 for each composition. All fibers were prepared at the same spinning speed. Although outside the scope of this work, others have examined the effect of fiber size on mechanical properties and found an inverse correlation with fiber size—smaller diameters generally yield improved modulus, strength, and toughness [22,23].

### 2.6. Thermal Analysis

DSC was used to understand the interaction of the polymers in the blends by examining changes in the glass transition and crystallization peaks (Table 2). Heat flow-temperature curves are displayed in the Supplementary Materials section (Figure S3). Shifts in these peaks indicate some level of miscibility of the polymers in the blends. PET and PP are both semi-crystalline polymers and exhibit crystallization peaks at 195.8 °C and 105.3 °C, respectively. The addition of PS and PP to PET shifted the crystallization peak to higher temperatures. The glass transition temperatures (T_g_s) were also shifted but remained separate, indicating partial miscibility of the blend. In the PP/PS blend, the T_g_s of both polymers were shifted 15–20 °C towards one another. For the ternary blend, the T_g_s remained similar to the neat polymers, suggesting immiscibility of the blend. The crystallization peak for PP was shifted by about 10 degrees but remained unchanged for PET. The relative crystallinity of PP blended with 50 wt % PS was reduced to 7.7%. The crystallinity of PP was further reduced by blending it with PET (1.4%). The crystallinity of PET was reduced from 35.4% to 17.2% and 20.5% with the addition of 50 wt % PP and PS, respectively. The ternary blend exhibited reduced crystallinity for both semi-crystalline polymers, with a value of 60% reduction for PET and 92% reduction for PP.

## 3. Discussion

In this work, we examined the feasibility of processing recycled PP, PS, and PET—three of the most common plastic wastes—into fibrous products. The fibers could have applications such as insulation, filtration media, and textiles. PET is known as a high performance polymer, possessing a high tensile strength and elastic modulus, while PP has low tensile properties. PS has mediocre tensile properties, and is a brittle polymer subject to fracture. While fibers can be used to make value-added products from these materials, high performance materials are not necessarily required to increase the value of the recycling stream. Insulation, bandages, wipes, and other disposable fabrics can be made from fibers with modest to mediocre properties. The incorporation of bottle-grade PET improved the tensile properties of the blends, but they are not close to the range of virgin materials (2 GPa elastic modulus for PET textile fibers vs. ca. 0.1 GPa for recycled PET fibers). However, work by Lee et al. suggests that they are not far from the range of both traditional melt spun rPET and virgin PET fibers (0.22–0.24 GPa), which suggests the mechanical property reduction is not due to the centrifugal spinning process [24]. Recycled polymers contain many contaminants and processing aids and have been potentially subjected to multiple thermal and mechanical stresses during processing cycles. In addition, the rPET shreds were not dried before processing, which could have contributed to molecular weight reduction due to hydrolysis in the melt. There could also be an effect of defect sensitivity in the micrometer sized fibers. These factors contribute to the reduced mechanical properties observed.

Crystallization can have a tremendous impact on fiber mechanical properties, and can also be affected by fiber composition. Wellen et al. found that the addition of even small amounts (1 wt %) of PS to PET reduced the rate of cold crystallization or crystallization upon heating due to the anti-nucleating effect of PS on PET, although the ultimate degree of crystallinity was not affected [25,26]. In our work, we found that the degree of crystallization was reduced by blending with 50 wt % PS. The addition of PP to PET cut PET’s degree of crystallization to about half, and the PP crystalline phase was reduced to almost zero (1.4%). This is contrary to most of the literature, which cites the nucleation effect of PET crystallites on PP crystallization [27,28]. However, Mirjalili et al. observed a decrease in PP crystallinity when blended with PET and a maleic anhydride grafted polypropylene compatibilizer [29]. Here, we did not use a compatibilizer, but the polymers were mixed in the melt at fairly high shear rates (6000–12,000 rpm) which may have imparted partial compatibility (or miscibility). In addition, certain additives in the polymers may have acted as compatibilizers. Partial miscibility is supported by the shifted T_g_s observed for both PP and PET. 

The onset temperature for crystallization can also be important for certain types of processing such as injection or blow molding. Optical clarity, barrier properties, and flexibility are requirements for beverage bottles. If the crystallinity is too high, stretching during the blow molding cycle may be impaired, while too low crystallinity can result in reduced barrier and mechanical properties. The addition of PS and displacement of the crystallization peak to higher temperatures can contribute to a wider range of cold crystallization phenomena from the segregation between PS and PET molecules [25,26]. The introduction of 1–20 wt % PS caused a shoulder on the crystallization peak in Wellen’s studies, indicative of at least two crystallization phenomena such as different crystalline geometries, but this was not observed in our studies. However, the crystallization peak increased ca. 4 °C with the addition of PS, in agreement with Wellen’s findings, indicating a small anti-nucleating effect of PS on PET. The addition of PP to PET followed similar trends to that of the PS/PET blend: the PET crystallization temperature increased and the PP crystallization temperature was unchanged. The addition of PS to PP had the opposite effect, and served to lower the onset crystallization temperature of PP by ca. 13 °C.

Yin et al. found that the repeated heating and processing of plastic wastes affects not only crystallization phenomenon, but also viscosity and mechanical properties. PP in particular can undergo chain scission after reprocessing (19 heating cycles in Yin’s work) or at temperatures above 270 °C [16]. Strain, stress, and energy to break were largely affected by the effective reduction in molecular weight. To combat this problem, blending of recycled plastics with virgin plastics has served to improve processability and mechanical properties. Yin blended recycled PP with 50 wt % virgin PP (Lyondell Basell) to produce fibers successfully used in the construction industry. The tensile strength was increased by ca. 28%. Another approach is to blend different recycled wastes [30]. There has been a fair amount of work examining polyolefin blends [31,32,33]. Ductility of PP and polyethylene (PE) blends increases with an increasing PE percentage, but tensile strength follows the opposite trend. Elongation at break dramatically decreases when 50 wt % PP is blended with either low-density or high-density polyethylene (LDPE, HDPE) due to the blend incompatibility. The addition of HDPE reduces the spherulite size of PP, decreasing the chance of fracture, but the poor interfacial adhesion limits the stress transfer [32].

Often, it is necessary to use compatibilizers which change the interface between the immiscible blend components. Luzuriaga et al. prepared LDPE–HDPE–PP–PS blends with poly(styrene-*co*-butadiene) block copolymers and a secondary-amine stabilizer which doubled the impact strength and improved the thermo-oxidative stability [34]. In our work, the addition of 50 wt % PET served to double the elastic modulus and tensile strength of the resulting fiber in most cases instead of using a compatibilizer. The addition of PP or PET to PS reduced brittleness and increased elongation to failure. The addition of PS to PP did not impact elongation to failure as PP/PS fibers stretched to ca. 70–80% before failure. This may be due to the small amount of PS actually incorporated into the spun fibers based on FTIR compositional analysis. However, the addition of either PP or PS to PET reduced elongation to 30% and 40%, respectively. The ternary blend fibers were quite brittle and failed at 20% elongation. However, they still had an improved modulus and tensile strength compared to neat recycled PS, PP, and the PP-containing blends. While the blends are largely immiscible, they have limited interactions which serve to affect the resulting ductility and strength of the fibers. The addition of PP to PET generally requires a compatibilizer and without it, mechanical properties and ductility are reduced. Polystyrene is a very brittle polymer and generally reduces ductility with the exception of blends with PP, which appears to have a minimal interaction based on DSC (minimal shift in T_g_).

The recycled PET and composite fibers of mixed waste streams, particularly those containing PET, are suitable for applications in insulation, composite reinforcement, and textiles. The use of compatibilizers such as thermoplastic maleic anhydride grafted copolymers and modified SBS block copolymers may further enhance their mechanical properties. Mixing the blends in a compounder prior to melt spinning could also provide more uniform blend compositions, as it is likely the polymers were not well mixed by grinding and in the brief time in the melt spinneret. The addition of viscosity-reducing additives may further reduce fiber diameters into the nano-meter range, opening up more applications including micro/ultra-filtration and insulation materials. 

## 4. Materials and Methods

For a source of recycled PET, plastic water bottles were obtained from an office recycle bin. The labels were removed, and the bottles were cut into strips with scissors, rinsed with water and ethanol, and allowed to dry. The PET strips were then shredded with a cross-cutting paper shredder. The recycled PS and PP materials were obtained from petri dishes and centrifuge tubes (respectively). Coarse particles were initially formed by processing the donor articles in a blender, followed by grinding in an Oster coffee grinder. Blends were prepared by mixing 50 wt % each of ground PS/PET, PS/PP, and PP/PET or 33 wt % each of PS, PP, and PET in a coffee grinder for 1 min. Two component blends reported in the figures are 50/50 wt % of the denoted polymers. Three component blends are 33/33/33 wt % of the denoted polymers.

Melt-spun fibers were prepared on a Fiberlab™ L1000-D from Fiberio^®^ by adding 100–300 mg of polymer to either a 20- or 30-G (orifice opening, three orifices per spinneret) 4 inch diameter melt spinneret. The polymer was heated with the upper and lower heaters until fully melted (ca. 230–300 °C, approximately 10–15 min). The collector consisted of 6-in high by ½-in wide bars in a circular pattern with a working distance fixed at 14 cm, as described in Zander et al. in 2015 [11]. The spinneret was rotated between 6000 and 12,000 rpm for 30 s to generate fibers on the collector and fully exhaust the polymer from the spinneret. 

The fiber morphology was probed using a field-emission scanning electron microscope (SEM, Hitachi S-4700, Hitachi, Tarrytown, NY, USA) after sputter coating with gold-palladium. Fiber diameters were measured using image analysis software (Image J v 1.34, National Institutes of Health, Bethesda, MD, USA). For each image, the “set scale” tool was utilized to determine the number of pixels in a given distance. Fibers were then measured manually using the measurement tool. The number of measurements for each specimen type varied from 96 to 699 data points.

Chemical analysis was performed by FTIR in attenuated total reflectance mode (Thermo Nicolet iS50, Thermo Fisher Scientific, Waltham, MA, USA) using 256 averaged scans and a 4 cm^−1^ resolution over a range of 4000–400 cm^−1^. 

Thermal properties were measured using differential scanning calorimetry (DSC) with a heat/cool/heat program (TA Instruments Q1000, New Castle, DE, USA). All samples were heated at 20 °C·min^−1^ to 300 °C, cooled 20 °C·min^−1^ to −50 °C, and then heated again at 20 °C·min^−1^ to 300 °C. DSC data was processed using Universal Analysis software. Two runs per specimen were carried out.

Rheological experiments were conducted on an AR2000 rheometer (TA Instruments, New Castle, DE, USA) at the spinning temperature for each respective polymer or blend. 25 mm aluminum plates were used. A shear rate of 1/s was applied with a ramp from 0.1 to 1000. A minimum of two runs per sample condition were collected. 

Uniaxial mechanical characterization was carried out using a Deben Microtensile stage (Deben, London, UK) with a 20 N load cell. Tests were run at a speed of 100 µm·min^−1^. Displacement was measured via the microtensile stage crosshead positioner (10 μm accuracy, 3 μm resolution) and the average strain was calculated based on the original length of the specimen. Gauge length was kept constant at 10 mm as a function of the tensile stage design. Tensile specimens were cut manually with a razor blade to dimensions of approximately 20 mm × 5 mm. Thickness *T* measurements of the mats were calculated from the mat weight and area. Elastic modulus was calculated from the initial slope of the stress-strain curve and the tensile strength was taken as the maximum of the engineering stress. 

## Figures and Tables

**Table 1:** Fiber diameter of recycled polymers at varied rotation speeds.

Polymer	rpm	Temperature (°C)	Gauge ^1^	Diameter (µm)	Standard Deviation (µm)	*n* ^2^
rPP	6000	220	20	5.98	2.93	187
	8000			3.93	2.38	185
	10,000			2.65	1.64	105
	12,000			1.92	0.87	96
rPP	6000	220	30	1.67	1.14	175
	8000			2.09	1.34	463
	10,000			1.90	1.20	447
	12,000			1.07	0.72	699
rPET	6000	300	20	9.41	4.25	252
	8000			5.84	3.07	353
	10,000			4.68	4.04	313
	12,000			4.56	2.97	377
rPS	6000	260	20	8.10	5.26	178
	8000			6.13	5.23	330
	10,000			4.74	3.07	329
	12,000			3.00	3.02	151

rPP = recycled polypropylene; rPET = polyethylene terephthalate; rPS = polystyrene; ^1^ spinneret orifice size; ^2^ number of fiber diameter data points.

**Table 2:** Thermal analysis of recycled bulk polymers and fibers prepared by centrifugal melt-spinning.

Sample	T_g_^1^ (°C)	T_c_^2^ (°C)	Relative Xc_PP_ (%)	Relative Xc_PET_ (%)
rPS	97.1	-	-	-
rPP	−17.0	105.3	31.0	-
rPET	62.6	195.8	-	35.4
PP/PS	−2.0/80.9	92.5	7.7	-
PP/PET	−8.4/51.7	103.8/203.8	1.4	17.2
PS/PET	95.7/74.9	200.4	-	20.5
PS/PP/PET	94.0/−20.3/65.34	94.4/198.4	2.5	14.1

^1^ Glass transition temperature; ^2^ Crystallization temperature.

## Supplementary Materials

The following are available online at www.mdpi.com/1996-1944/10/9/1044/s1, Figure S1: Full range FTIR spectra of recycled polymers and blends (50/50 wt % and 33/33/33 wt %); Figure S2: Representative load-displacement curves of recycled polymers and blends (50/50 wt % and 33/33/33 wt %); Figure S3: Heat-flow-temperature curves of recycled polymers and blends (50/50 wt % and 33/33/33 wt %).
