# Supplementary material for: Composite Fibers from Recycled Plastics Using Melt Centrifugal Spinning
Source: Materials (Basel). 2017 Sep 6;10(9):1044. doi: 10.3390/ma10091044 (PMC5615699; doi:10.3390/ma10091044)
Supplement: Supplementary file 1 [file materials-10-01044-s001.pdf]

Supplementary

Article

Composite Fibers from Recycled Plastics Using Melt Centrifugal Spinning

Nicole E. Zander *, Margaret Gillan and Daniel Sweetser

United States Army Research Laboratory, Weapons and Materials Research Directorate, Aberdeen Proving Ground, Aberdeen, MD 21005, USA; margaret.gillan2.ctr@mail.mil (M.G.); sweetser@udel.edu (D.S.)

* Correspondence: nicole.e.zander.civ@mail.mil; Tel.: +1-410-306-1965

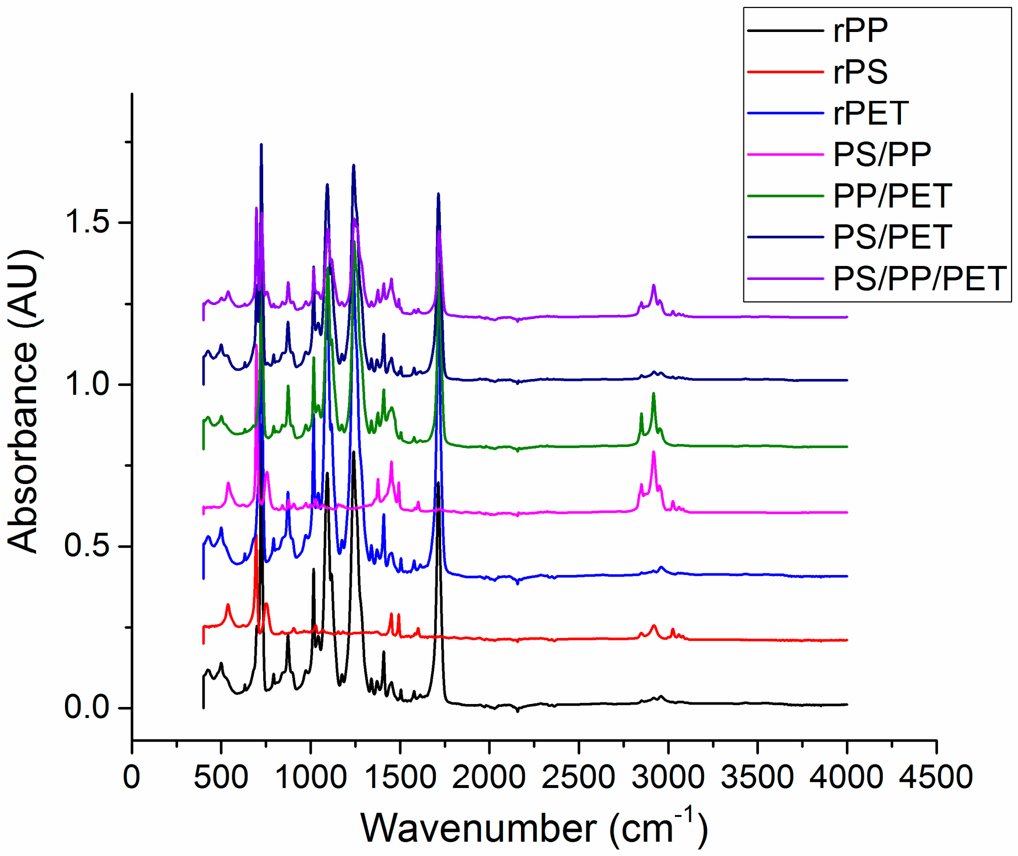

Figure S1: Full range FTIR spectra of recycled polymers and blends (50/50 wt %and 33/33/33 wt %)

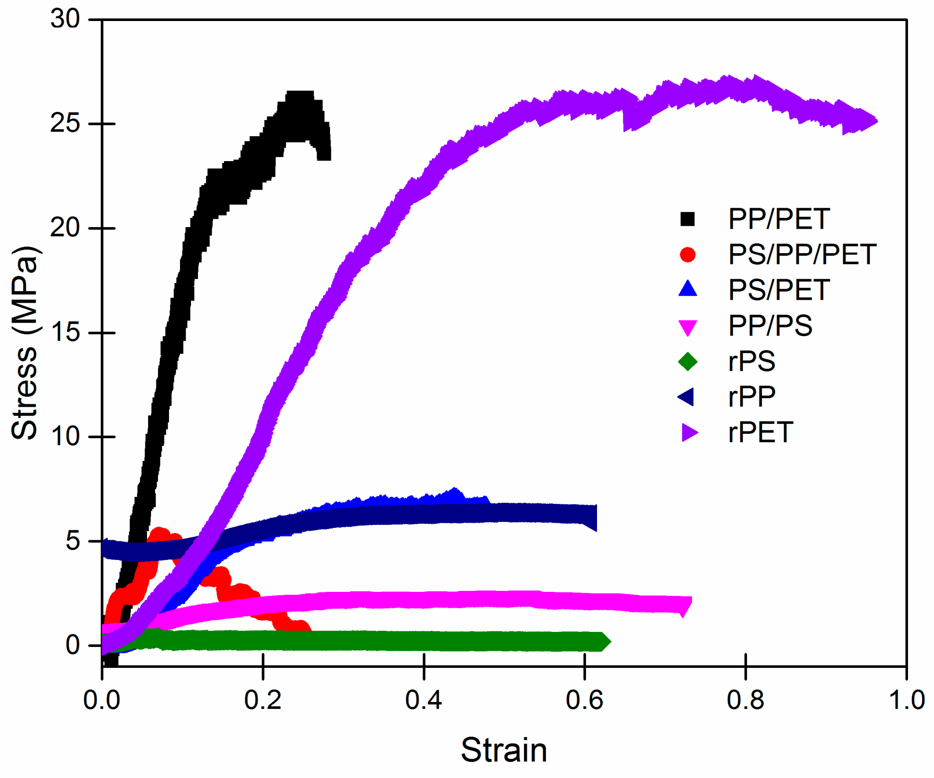

Figure S2: Representative load-displacement curves of recycled polymers and blends (50/50 wt % and 33/33/33 wt %)

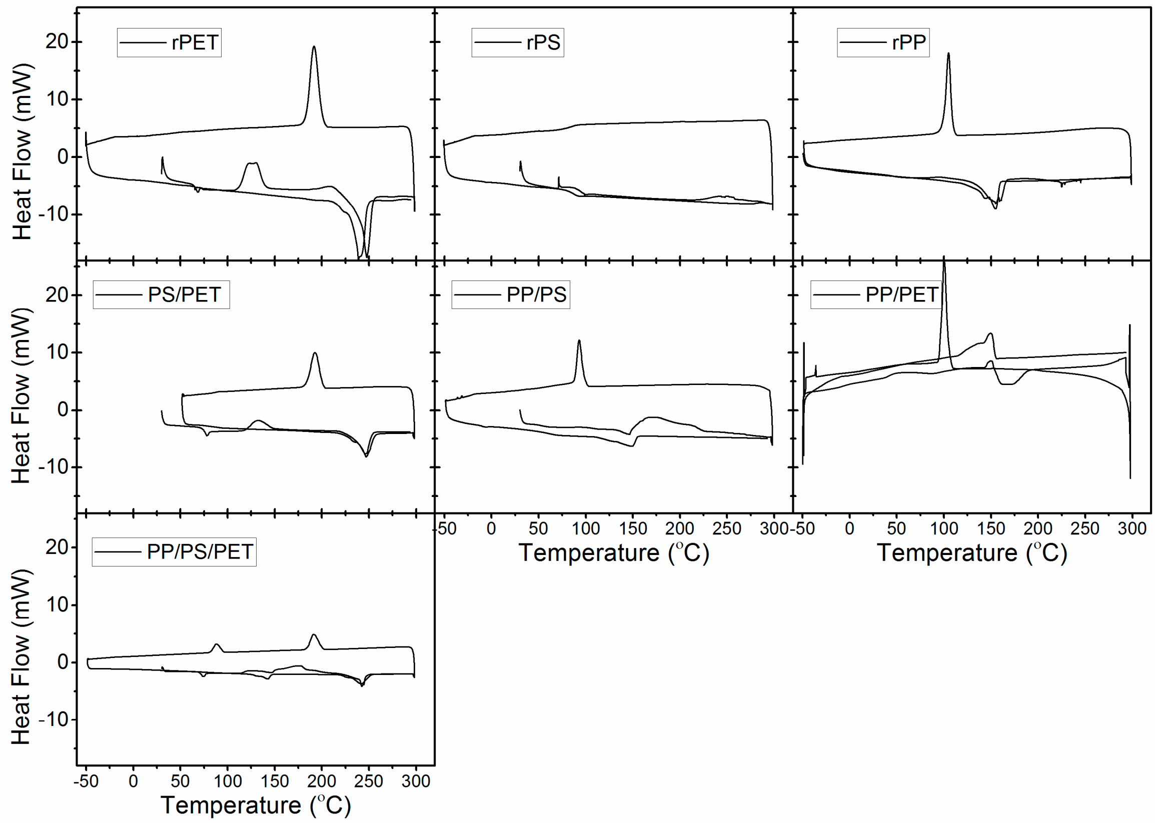

Figure S3: Heat-flow-temperature curves of recycled polymers and blends (50/50 wt % and 33/33/33 wt %)
